# Phagentherapie – Mit Viren gegen Bakterien

**DOI:** 10.1007/s00103-025-04064-y

**Published:** 2025-06-02

**Authors:** Timo Faltus, Christian Willy

**Affiliations:** 1 Juristische und Wirtschaftswissenschaftliche Fakultät, Juristischer Bereich, Martin-Luther-Universität Halle-Wittenberg, Universitätsplatz 3–5, 06099 Halle (S.), Deutschland; 2 Klinik für Unfallchirurgie und Orthopädie, Septisch-Rekonstruktive Chirurgie, Forschungs- und Behandlungszentrum Rekonstruktion von Defektwunden, Bundeswehrkrankenhaus Berlin, Scharnhorststraße 13, 10115 Berlin, Deutschland

Die Zunahme multiresistenter Bakterien (Multiantibiotikaresistenz) stellt eine der größten aktuellen Bedrohungen für die öffentliche Gesundheit dar. Bereits vor 10 Jahren wurden für Deutschland etwa 30.000 bis 35.000 Infektionen mit einem multiresistenten Erreger und 1000 bis 4000 darauf zurückführbare Todesfälle berechnet [1]. Für das Jahr 2021 geht das Institute for Health Metrics and Evaluation (IHME) von mehr als 8000 direkt auf Infektionen mit multiresistenten Bakterien zurückführbaren Todesfällen in Deutschland aus [2]. EU/EWR-weit wurden für das Jahr 2019 mehr als 850.000 Infektionen mit antibiotikaresistenten Erregern geschätzt, verbunden mit ca. 38.000 Todesfällen [3]. Konventionelle Antibiotika verlieren zusehends an Wirksamkeit, während die Entwicklung neuer Antibiotika nicht Schritt halten kann. Die Suche nach alternativen Therapieansätzen rückt daher in den Fokus von Medizin und Gesundheitspolitik. In Anbetracht der alarmierenden Zahlen forderten schon die G7 (2015; [4, 5]) und die G20 (2018; [6]) verstärkte Maßnahmen gegen antimikrobielle Resistenzen.

Eine Alternative zur Verwendung von Antibiotika ist die Bakteriophagentherapie. Bakteriophagen (Phagen) sind Viren, die Bakterien, nicht aber menschliche Zellen infizieren, lysieren und dadurch abtöten, wodurch die Ursache einer bakteriellen Infektion behandelt werden kann. Phagen können daher dort wirken, wo Antibiotika zunehmend versagen – und das, ohne menschliche Zellen anzugreifen und ohne – anders als Antibiotika – das gesunde Mikrobiom zu schädigen.

Bereits vor über 100 Jahren – vermutlich 1919 [7, 8] – wurde die Phagentherapie durch Felix d’Herelle in Paris am Hôspital des Enfants Malades erfolgreich zur Behandlung bakterieller Infektionen angewandt. In den 1930er/1940er-Jahren erzielte dieser Ansatz auch in Deutschland vielversprechende Ergebnisse [9, 10]. Mit dem Siegeszug der Antibiotika nach dem Zweiten Weltkrieg geriet die Phagentherapie in Westeuropa allerdings nahezu in Vergessenheit und wurde jahrzehntelang kaum weiterentwickelt. Demgegenüber wurde die Therapieform in Teilen Osteuropas (insbesondere in Ländern der ehemaligen Sowjetunion, wie z. B. Georgien) kontinuierlich erforscht und angewendet. Heute erlebt die Phagentherapie auch in den westlichen Industrienationen eine Renaissance. Insbesondere bei Infektionen mit multiresistenten Keimen gilt die Phagentherapie wieder als vielversprechende Behandlungsoption. Darauf weist auch das im Jahr 2021 vom Deutschen Bundestag beauftragte Gutachten zum zukünftigen Stellenwert der Phagentherapie hin [11].

In den westeuropäischen Ländern ist die Phagentherapie allerdings bislang keine etablierte Therapieoption. In der Europäischen Union und in Deutschland fehlen nach EU-Recht bzw. nach deutschem Recht zugelassene Phagenarzneimittel. Nur in der Slowakei und Tschechien ist mit Stafal® ein bereits vor dem EU-Beitritt beider Länder zugelassenes Phagenarzneimittel auf dem Markt. In Deutschland werden Phagen heute zumindest im Rahmen individueller Heilversuche eingesetzt, insbesondere wenn Antibiotika nicht zur Heilung geführt haben. Auch wenn die bisherigen Ergebnisse dieser individuellen Anwendungen und der Erforschung der Phagentherapie für eine hohe Patientensicherheit und gute Verträglichkeit sprechen, existieren bislang nicht ausreichend evidenzbasierte klinische Daten für die Routineanwendung, insbesondere liegen nur vereinzelte Ergebnisse aus randomisierten kontrollierten Studien vor, die als Goldstandard für den Wirksamkeitsnachweis gelten.

Die wesentliche technische Herausforderung der Translation der Phagentherapie in die medizinische Routine ist die Wirtsspezifität der Phagen. Die Mehrheit der für die medizinische Verwendung relevanten Phagen infiziert nur einzelne Bakterienstämme einer Bakterienspezies. Heute wird der überwiegende Teil der Phagentherapeutika nach Identifikation des bakteriellen Erregers auf Abruf spezifisch für den jeweiligen Patienten hergestellt. Typischerweise findet diese individuelle Herstellung bei stationärer Behandlung auf ärztliche Verschreibung in der Krankenhausapotheke statt (sogenannte magistrale Herstellung) und bedarf keiner arzneimittelrechtlichen Zulassung. Bestimmte polyvalente Phagen können eine höhere Anzahl von Bakterienstämmen infizieren. Auf Basis dieser Phagen können daher statische, patientenunabhängige Arzneimittel vorab hergestellt werden, die wie andere patientenunabhängig und im Voraus hergestellte Arzneimittel ein Zulassungsverfahren durchlaufen müssen. Sowohl die patientenspezifische als auch die patientenunabhängige Variante der Phagentherapie weisen heute aber noch technische, ökonomische und rechtliche Herausforderungen auf, die die Zugänglichkeit der Phagentherapie derzeit begrenzen.

Die heutigen Translationsbemühungen der Phagentherapie finden im Spannungsfeld der benötigten Bereitstellung der Phagen und der Erzeugung klinischer Evidenz statt. Hierzu muss insbesondere die Bereitstellung der Phagen technisch weiterentwickelt werden, um die klinische Anwendung zu standardisieren. Zum anderen muss geprüft werden, inwieweit der regulatorische Rahmen die Phagentherapie angemessen abbildet. Das heutige Arzneimittelrecht ist auf die Entwicklung und Bereitstellung von patientenunabhängigen Arzneimitteln ausgerichtet. Die patientenindividuelle, apothekenbasierte Herstellung auf Abruf ist zwar zulässig. Ungeklärt ist aber, ob die jeweiligen Vorschriften eine valide Basis für die Routineanwendung individueller Phagentherapeutika wären.

In jüngster Zeit gibt es Entwicklungen, die die Rahmenbedingungen für zumindest einzelne Aspekte der Phagentherapie verbessern könnten. So hat die Europäische Arzneimittel-Agentur (EMA) im Dezember 2023 den Entwurf des Konzepts für eine Leitlinie für die Entwicklung und Herstellung von Phagenarzneimitteln veröffentlicht. Der Konsultationsprozess für diesen Entwurf endete im März 2024 [12]. Im Europäischen Arzneibuch (Pharmacopoeia Europaea, Ph. Eur.), herausgegeben vom Europäischen Direktorat für die Qualität von Arzneimitteln (EDQM), ist mit der im Juli 2024 erfolgten Veröffentlichung des 11.6 Supplements erstmals das allgemeine Kapitel 5.31 zu Phagenarzneimitteln enthalten, womit technische Anforderungen an die Herstellung und Kontrolle von Phagenarzneimitteln beschrieben werden [13]. Parallel dazu entsteht in Deutschland unter Federführung der Arbeitsgemeinschaft der Wissenschaftlichen Medizinischen Fachgesellschaften e.V. (AWMF) eine erste klinische Behandlungsleitlinie (AWMF S2k) zur Phagentherapie, die bis Juli 2025 erwartet wird [14].

100 Jahre nach den ersten Phagenbehandlungen nehmen die technischen und regulatorischen Voraussetzungen konkrete(re) Formen an. Dieses Themenheft erscheint damit zu einem Zeitpunkt, in dem die Realisierung der Phagentherapie in Anbetracht der eingangs genannten zunehmenden Antibiotikaresistenzen (AMR) wieder an Fahrt gewinnt. Hierbei schildern die ersten beiden Beiträge des Themenhefts zum einen die gesundheitsökonomische Bedeutung antimikrobieller Resistenzen, zum anderen werden Strategien zur Eindämmung dieser Resistenzen beschrieben. Darauf aufbauend stellt das Themenheft den aktuellen Stand der Phagentherapie medizinisch, gesundheitspolitisch, rechtlich, ethisch und ökonomisch dar und gibt Hinweise für die zukünftige Entwicklung. Die Beiträge dieses Themenheftes unterstreichen, dass die Wiederbelebung der Phagentherapie in Deutschland eine vielschichtige Aufgabe ist. Die Bedrohung durch zunehmende Multiantibiotikaresistenzen erfordert entschlossenes Handeln und innovative Lösungen – Bakteriophagen können hierbei eine entscheidende Rolle spielen, jedoch nur im Zusammenspiel mit wissenschaftlicher Evidenz, klaren regulatorischen Strukturen und verantwortungsvoller Anwendung. Die Palette der hier versammelten Artikel macht deutlich, dass interdisziplinäre Zusammenarbeit der Schlüssel ist, um die Phagentherapie aus der Nische in die Regelversorgung zu führen. Gelingt dies, könnten Phagen im Zeitalter der Multiantibiotikaresistenz zu einer dringend benötigten Ergänzung unserer therapeutischen Optionen werden.

Die Realisierung dieses Themenhefts hat Professor Dr. Eberhard Hildt (Paul-Ehrlich-Institut/Hasso Plattner-Institut Digital Health Cluster) als Mitglied des Herausgeberbeirats des Bundesgesundheitsblattes unterstützt, wofür wir uns herzlich bedanken.
